# Supplementary material for: Towards unified AI-driven fracture mechanics: the extended deep energy method (XDEM)
Source: Nat Commun. 2026 Aug 15;17:8492. doi: 10.1038/s41467-026-76748-1 (PMC13484471; doi:10.1038/s41467-026-76748-1)
Supplement: Supplementary file 3 [file 41467_2026_76748_MOESM3_ESM.zip › pic/Method/parametered_transfer.pdf]

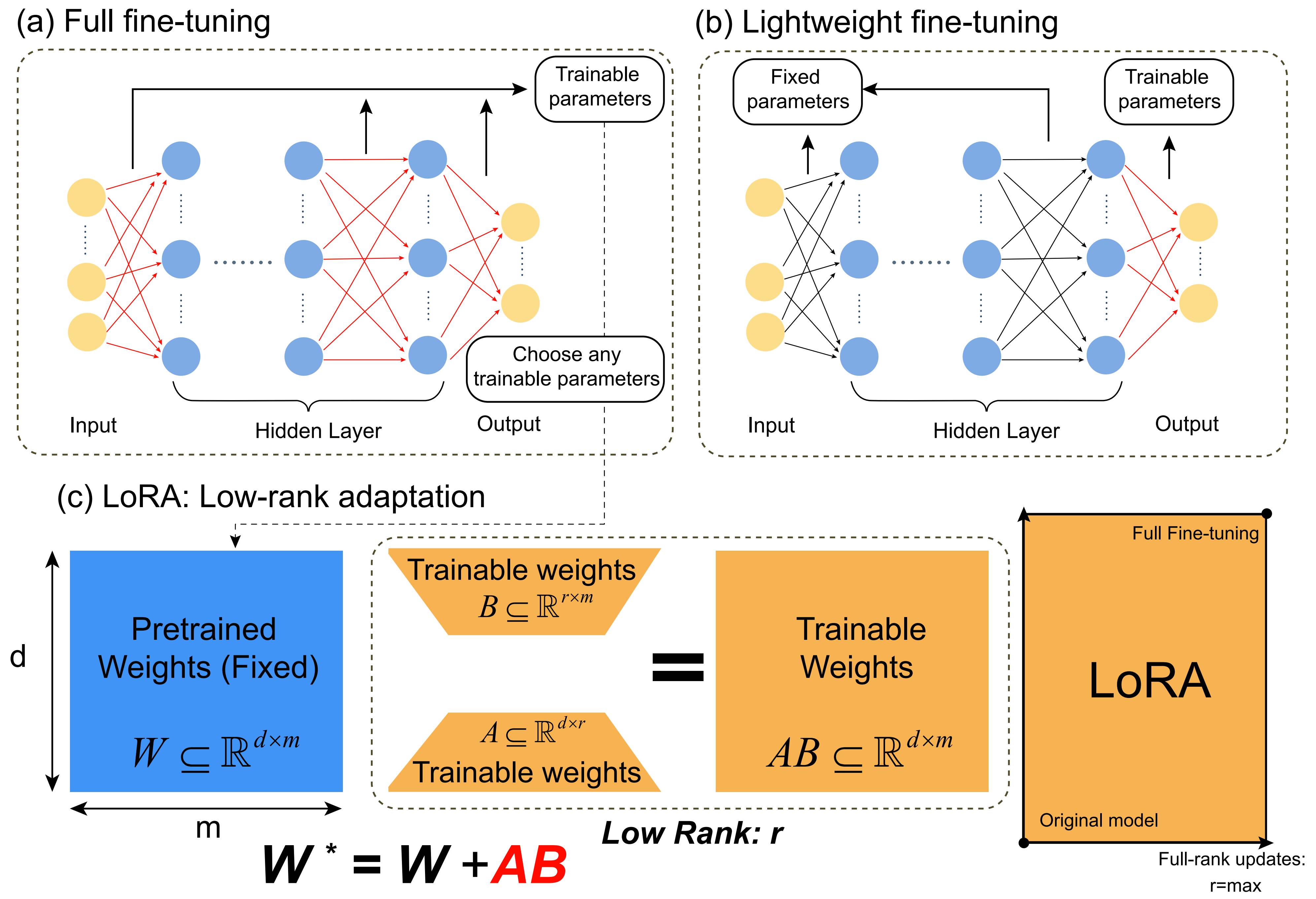
(a) Full fine-tuning
Trainable parameters
Choose any trainable parameters
Input
Hidden Layer
Output
(b) Lightweight fine-tuning
Fixed parameters
Trainable parameters
Input
Hidden Layer
Output
(c) LoRA: Low-rank adaptation
d
Pretrained Weights (Fixed)
$W \subseteq \mathbb{R}^{d\times m}$
m
Trainable weights
$B \subseteq \mathbb{R}^{r\times m}$
$A \subseteq \mathbb{R}^{d\times r}$
Trainable weights
Trainable Weights
$AB \subseteq \mathbb{R}^{d\times m}$
Low Rank: r
$W^* = W + AB$
Full Fine-tuning
LoRA
Original model
Full-rank updates: r=max
